# A homotrimeric GPCR architecture of the human cytomegalovirus revealed by cryo-EM

**DOI:** 10.1038/s41421-024-00684-x

**Published:** 2024-05-16

**Authors:** Yanyan Chen, Yang Li, Qingtong Zhou, Zhaotong Cong, Shi Lin, Jiahui Yan, Xianyue Chen, Dehua Yang, Tianlei Ying, Ming-Wei Wang

**Affiliations:** 1 Research Center for Deepsea Bioresources, Sanya, Hainan China; 2 Shanghai Institute of Infectious Disease and Biosecurity, Department of Medical Microbiology and Parasitology, School of Basic Medical Sciences, Fudan University, Shanghai, China; 3 Department of Pharmacology, School of Basic Medical Sciences, Fudan University, Shanghai, China; 4 The National Center for Drug Screening, Shanghai Institute of Materia Medica, Chinese Academy of Sciences, Shanghai, China; 5 Department of Chemistry, School of Science, The University of Tokyo, Tokyo, Japan; 6 School of Pharmacy, Hainan Medical University, Haikou, Hainan China

**Keywords:** Cryoelectron microscopy, Molecular biology

Dear Editor,

Herpesviruses encode G protein-coupled receptors (GPCRs) in their viral genomes and express these receptors in infected host cells to reprogram cell signaling networks of the host for survival, replication and pathogenesis. As a ubiquitous herpesvirus with a seroprevalence of more than 50% in adults, human cytomegalovirus (HCMV) encodes four viral GPCRs (vGPCRs) including US27, US28, UL33 and UL78. The first three show about 30% homology to chemokine receptors (e.g., CXCR3, CX3CR1, CCR10 and CXCR1)^1^, whereas UL78 displays negligible homology to any endogenous receptors (Supplementary Figs. S1, S2)^2^. US28 is not only constitutively active but also capable of silencing the signal transduction of chemokine receptors by binding to various chemokines and promiscuously coupling to G proteins^3^. Meanwhile, four X-ray and three cryo-electron microscopy (cryo-EM) structures have been determined for US28 in the presence of either structural ligand (CX3CL3 or CX3CL1.35), intracellular binder (nanobody or G protein) or both; all of them adopt active or active-like conformations (Supplementary Table S1)^4,5^. While previous studies suggest that US27, UL33 and UL78 regulate viral replication or reactivation, there is no existing evidence demonstrating their interactions with chemokines; thus, they are classified as orphan receptors^5,6^. Among the two reported cryo-EM structures, US27 has an occluded ligand-binding pocket and captures a guanosine diphosphate-bound inactive G_i_ (Supplementary Table S1)^5^. Similarly, the inhibitory G protein (G_i_)-coupled Epstein-Barr virus (EBV)-encoded GPCR (BILF1) was found to adopt an occluded extracellular surface to block the typical chemokine-binding site^7^.

Besides the traditional vGPCR-mediated signaling paradigms including ligand-dependent signaling through a ligand−receptor−effector complex (as seen in US28) or ligand-independent, constitutive signaling via a receptor−effector complex (observed in US27 and BILF1), receptor hetero- or homo-oligomerization has gained increasing experimental supports^4,8^. Notably, UL78 and UL33 can either form heterodimers with human CXCR4 and CCR5 interrupting normal signaling, or present oligomers to modulate the functions of other vGPCRs and host GPCRs^9^. Moreover, several chemokine receptors exhibit features of dimerization and higher order oligomerization^9–11^. Atomic visualization of GPCR oligomerization has been achieved mainly in classes C and D1 GPCR dimers, except one class A GPCR (apelin receptor) dimer^12^. In this study, we report the cryo-EM structure of UL78 and unveil a novel form of GPCR oligomerization unseen before — a homotrimeric architecture, thereby broadening our knowledge about GPCR structures.

To determine the cryo-EM structure of UL78, we followed the protocols previously adopted in solving the structures of US27 and BILF1^5,7^, wherein the receptor was co-expressed with G_i_ protein, and the formation of the UL78−G_i_ complex was confirmed by size exclusion chromatography and SDS-PAGE. However, only a small fraction of the complex particles appeared in 2D classification (Fig. 1a; Supplementary Fig. S3a), and most of them were observed with a clear trimeric architecture, unaffected by the absence of G_i_ protein or the fused LgBiT (Supplementary Fig. S3b, c). When the receptor was expressed solely, only the trimeric form rather than monomer was detected in non-denaturing gel (Supplementary Fig. S3c). After sample preparation, cryo-EM data collection and single-particle analysis, a 3D consensus density map was reconstructed with a global resolution of 3.12 Å (Fig. 1b; Supplementary Fig. S4), enabling model building for all seven transmembrane helices (TMs), three intracellular loops (ICLs) and extracellular loops (ECLs) 1 and 2 (Supplementary Fig. S5 and Table S2).

**Fig. 1:**
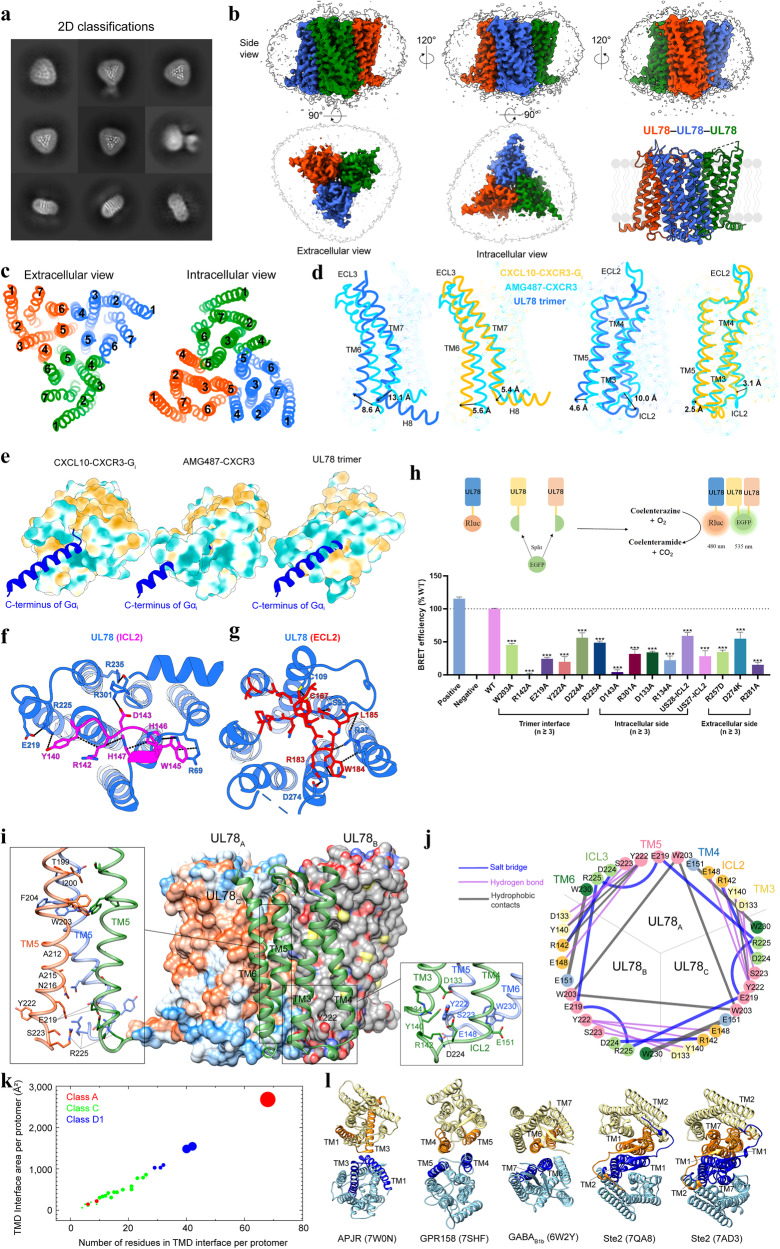
Cryo-EM structure of homotrimeric UL78. **a** Representative cryo-EM 2D classification averages of UL78 homotrimers. **b** Cryo-EM density map and cartoon representation of UL78 homotrimer. The sharpened cryo-EM density map at the 0.23 threshold shown as a light gray surface indicates a micelle diameter of 10 nm. **c** Extracellular and intracellular views of TMs show the interfaces of UL78 trimer. **d** Structural comparison with the inactive-state CXCR3 structure (PDB: 8K2W) shows conformational changes of TMs 5–7 and ICL2 of UL78. **e** Steric clashes between the G_i_ α5 helix (extracted from the aligned CXCL10-bound CXCR3−G_i_, PDB: 8K2X) and the intracellular faces of inactive CXCR3 (PDB: 8K2W) and UL78. **f** Magnified view of ICL2 within the intracellular pocket and its interactions with TMs and ICL1. **g** Magnified view of ECL2 within the extracellular pocket and its interactions with TMs. **h** Detection of UL78 trimer formation by BiFC-BRET assay. Data are means ± SEM from at least three independent experiments. All data were analyzed by one-way ANOVA and Dunnett’s test, ***P < 0.001. Additional mutations around the ECL2 (C109^TM3^A, W184^ECL2^A, Q188^ECL2^A and R254^TM6^A) consistently decreased the trimer formation by 52%, 60%, 56% and 49%, respectively. **i** Homotrimer interface of UL78, where two protomers are displayed in surface representation, and one in cartoon. Magnified views of the tightly packed TM5 from three protomers (left) and the intracellular bottom of the trimer interface (right) are shown. **j** Interface interactions among three UL78 protomers. The colors of the circles and lines are proportional to the residue positions and the interaction types, respectively. **k** Scatter plot of the TMD interface in GPCRs. The *y* axis represents the TMD interface area per protomer, and the *x* axis is the number of residues from individual protomers in the TMD interface. The former is calculated using freeSASA, while the latter is counted once the residual interface area is over 1 Å^2^. PDBs with multiple protomers are averaged in the plot. The circles are colored according to GPCR subfamilies and their diameters are proportional to the number of TMs in the interface. **l** Representative TM packings in GPCR dimer structures. The TMs in the interface are colored in orange and blue, respectively. Only the TMDs are shown for clarity.

The overall structure of UL78 comprises a homotrimer along the 3-fold symmetry axis with each protomer closely interacting with the other two (Fig. 1c). Three protomer structures share a highly conserved conformation with Cα root mean square deviation (RMSD) values of 0.50–0.56 Å and adopt a unique conformation that distinct from both active and inactive CXCR3 structures (Cα RMSD values of 1.06 Å and 1.28 Å, respectively) (Supplementary Fig. S6).

Specifically, the inward movements (10.0 Å and 13.1 Å) of ICL2 and the intracellular end of TM7 fulfill the receptor intracellular crevice (Fig. 1d), thereby hindering the insertion of the C-terminus of Gα_i_ (Fig. 1e–h). This intracellular closure is achieved by various interactions, including D133^TM3^−R134^TM3^−E148^ICL2^ salt bridges, D143^ICL2^−R301^H8^ salt bridge, R69^ICL1^−W145^ICL2^−H146^ICL2^−H147^ICL2^−R142^ICL2^ stackings, F127^TM3^−F239^TM6^−Y240^TM6^−F294^TM7^−F298^TM7^ stackings and I138^TM3^−V229^TM6^ hydrophobic contacts (Fig. 1f). Consistently, alanine mutations at D133^ICL2^, R134^ICL2^, D143^ICL2^ and R301^H8^ decreased the bimolecular fluorescence complementation (BiFC)−bioluminescence resonance energy transfer (BRET)-measured trimer formation by 66%, 78%, 96% and 68%, respectively, while replacement of UL78 ICL2 by those of US28 and US27 caused notable reduction in BRET signal by 41% and 72%, respectively, indicating a unique conformation of ICL2 favoring the UL78 trimer formation (Fig. 1h). By comparison, the inactive CXCR3 and many other class A GPCRs prefer the intracellular part of TM6 to tightly pack TM3 and then move outward to accommodate G protein binding (Fig. 1d, e).

Looking at the extracellular side, the top of orthosteric ligand-binding pocket was significantly covered by the inward-folded ECL2, while the latter forms many polar contacts with the surrounding TMs (Fig. 1g; Supplementary Fig. S7). A series of charged residues (R183^ECL2^, D274^TM7^, R257^TM6^, E277^TM7^ and R254^TM6^) along with polar residues including Q188^ECL2^ and Y250^TM6^, contribute profound interactions to stabilize the ECL2 coverage. Additionally, the orthosteric ligand-binding pocket was narrowed by two inter-TM residue pairs, including S95 in TM2 and R281 in TM7 via one hydrogen bond, and D117 and Y121 in TM3 and D197 and K201 in TM5 via multiple polar contacts. Two mutations (R257^TM6^D and R281^TM7^A) decreased the trimer formation by ~65% and 85%, respectively (Fig. 1h). Despite the same β-hairpin conformation, ECL2 of UL78 differs from those of CXCR3 and US28 in both apo and chemokine-binding states (Supplementary Fig. S7), whose extracellular vestibules are open and accessible for chemokine recognition. Although the ECL2 of BILF1 and US27 also forms a lid that caps the chemokine-binding pocket, the extracellular architecture of UL78 is more compact, and lacks the participation of ECL3 or interaction with the N-terminus (Supplementary Fig. S7). These structural features highlight a unique extracellular conformation of UL78.

UL78 forms a stable homotrimeric architecture through an extensive interface covering TMs 3–6, ICL1 and ICL2 with a large interface area (7956 Å^2^) (Fig. 1i–k; Supplementary Fig. S8). A central triangle in the trimer was formed by the tightly packed TM5 from three protomers, while the extracellular and intracellular halves of TM5 were further clasped by TM4 and TM3 from one neighboring protomer, respectively (Fig. 1j; Supplementary Fig. S9). Additionally, TM6 stacks in parallel with TM4 from the adjacent protomer. To stabilize the trimer interface, the extracellular segments of TMs made massive hydrophobic and stacking interactions especially through six adjacent aromatic residues (W203^TM5^−F204^TM5^ from three protomers) (Fig. 1i, j). In comparison, polar interactions were more dominant in the intracellular bottom of the trimer interface, where R142^ICL2^ has one salt bridge with D224^ICL3^ of the adjacent protomer, R225^ICL3^ forms two salt bridges with the downward E219^TM5^ and one hydrogen bond with S223^TM5^, while Y222^TM5^ penetrates into the TM3−TM4 cleft of the adjacent protomer forming two hydrogen bonds with D133^TM3^ and E148^ICL2^ (Fig. 1j). These observations are consistent with the BiFC−BRET assay results showing that R142^ICL2^A, E219^TM5^A, W203^TM5^A, Y222^TM5^A, D224^ICL3^A and R225^ICL3^A decreased the trimer formation by 98%, 76%, 54%, 80%, 44% and 51%, respectively (Fig. 1h). Such a unique homotrimeric architecture of UL78 employs a maximal number of residues (68 positions) and four TMs to construct the largest transmembrane domain (TMD) interface area per protomer (2680 Å^2^) among these reported GPCR structures^12–14^, including active and inactive Ste2 (fungal class D1 GPCR), classes A and C GPCRs (Fig. 1k, l).

Taken together, using cryo-EM and available structures, we have discovered a homotrimeric architecture of GPCR. The homotrimeric interface of UL78 differs significantly from all reported GPCR homodimer or heterodimer structures and illuminates a new and most compact TMD packing arrangement. Meanwhile, the special conformations of UL78 at both intracellular and extracellular sides may occupy the orthosteric pocket and impede the access of signaling proteins, supporting the hypothesis that UL78 is not a conventional ‘orphan’ receptor and acts via receptor oligomerization. Considering its trafficking between cell surface and cytoplasm^15^ and its ability to heterodimerize with US28, CCR5 and CXCR4, both structural and functional studies on the heteromeric UL78 with US28/CCR5/CXCR4 in the presence or absence of G proteins are worth pursuing^9^. Clearly, further exploration of the physiological significance of our discovery could expand the knowledge about GPCR biology.

### Supplementary information

Supplementary Information

## Data Availability

The atomic coordinates and the electron microscopy maps of the UL78 trimer have been deposited in the Protein Data Bank (PDB) under accession code 8Z1E, and Electron Microscopy Data Bank (EMDB) under accession code EMD-39724.
