# Supplementary material for: A homotrimeric GPCR architecture of the human cytomegalovirus revealed by cryo-EM
Source: Cell Discov. 2024 May 16;10:52. doi: 10.1038/s41421-024-00684-x (PMC11096299; doi:10.1038/s41421-024-00684-x)
Supplement: Supplementary file 1 — Supplementary Information [file 41421_2024_684_MOESM1_ESM.pdf]

# Supplementary Information

## Materials and Methods

### Cell culture

*Spodoptera frugiperda* (*Sf*9) and HighFive insect cells (Expression Systems) were grown in ESF 921 serum-free medium (Expression Systems) at 27°C and 120 rpm.

### Expression and purification of UL78

The full-length UL78 was cloned into the pFastBac vector and modified with the native signal sequences replaced by the HA signal peptide to facilitate receptor expression. To improve the stability of UL78−$G_i$ complex, the C terminus of UL78 was directly attached to LgBiT subunit. After the co-expression by infecting *Sf*9 cells (the ratio of FLAG-UL78-LgBiT, $G_i$, Gβ1-HiBiT and Gγ was 1:2:2:2), the complex was purified by the FLAG tag on the N terminus of UL78. Briefly, cell pellets were lysed in a buffer containing 20 mM HEPES pH 7.4, 100 mM NaCl supplemented with EDTA-free protease inhibitor cocktail (TargetMol). The complexes were formed by adding 15 μg/mL ScFv16 and 25 mU/mL apyrase (New England Biolabs). The complexes were then solubilized from membrane using 0.5% (w/v) lauryl maltose neopentyl glycol (LMNG, Anatrace), 0.03% (w/v) cholesteryl hemisuccinate (CHS, Anatrace) for 3 h at 4°C. Supernatant was collected by centrifugation at 65,000 × *g* for 30 min at 4°C, and taken to bind with Anti-DYKDDDDK G1 Affinity Resin (Genscript) overnight at 4°C. After packing, the resin was washed with 20 column volumes (CVs) buffer containing 20 mM HEPES pH 7.4, 100 mM NaCl, 10% (v/v) glycerol, 25 μM TCEP, 5 mM $MgCl_2$, 0.03% (w/v) LMNG, 0.01% (w/v) GDN and 0.008% (w/v) CHS. The complex was eluted from the resin by competitive elution and concentrated to 500 μL using a 100 kDa MWCO Amicon Ultra Centrifugal Filter. Size exclusion chromatography was carried out by loading the protein sample to Superdex 200 Increase 10/300GL (Cytiva). The column was preequilibrated with 20 mM HEPES, pH 7.4, 100 mM NaCl, 100 μM TCEP, 2 mM $MgCl_2$, 0.00075% (w/v) LMNG, 0.00025% (w/v) GDN and 0.00015% (w/v) CHS. The separated samples were concentrated to 8 mg/mL for cryo-EM analysis.

### Cryo-EM data acquisition

The cryo-EM sample was prepared by plunge vitrification in liquid ethane on a Vitrobot Mark IV (ThermoFisher Scientific) with blotting chamber set to 4°C and 100% humidity. Briefly, 3.6 μL sample solution was applied to glow-discharged holey carbon grids (Quantifoil, R1.2/1.3, Au 300 mesh) and blotted for 5 s before plunging. The data were collected on a Titan Krios (ThermoFisher Scientific) 300

kV electron microscope equipped with a Gatan K3 Summit direct electron detector, and serial EM3.7 was used to acquire cryo-EM images. The microscope was operated at a nominal magnification of 46,685× in counting mode, corresponding to a pixel size of 1.071 Å. The total exposure time was set to 7.2 s with intermediate frames recorded every 0.2 s, resulting in an accumulated dose of 80 electrons per $Å^2$ fractionated into a movie stack of 36 frames with defocus range of −1.2 to −2.2 μm. Totally, 5,527 movies were collected.

**Cryo-EM data processing**

The collected data were processed by cryoSPARC (v.4.2.1) and summarized in Supplementary Fig. S4. The micrographs underwent motion correction using patch motion correction and patch CTF estimation. Micrographs under 5 Å CTF resolution were cut off by Curate Exposures. Particles were auto picked by "Blob picker" using a particle diameter of 280 Å. Particle images were extracted with a box size of 256 pixel. After reference-free 2D classification, selected class averages were used for template-based particle picking. A total of 4,215,062 particle projections were subjected to two rounds of 2D classification to discard false positive particles or particles categorized in poorly defined classes, producing 2,424,875 particle projections for further processing. After another round of 2D classification to remove poor resolution particles, three 3D reference models of particle images were generated by *ab initio* reconstruction, and heterogeneous refinement was applied to divide particles into three subsets, and one subset with a well-ordered structure cryo-EM map was subjected to further 3D auto-refinement with a mask on the homotrimer. A dataset of 1,013,019 particles was subjected to 3D refinement, non-uniform refinement and local refinement, resulting in one well-defined subset with 98,594 projections. After the last round of refinement, the resulting cryo-EM map has a global nominal resolution of 3.35 Å by the 0.143 criteria of the gold-standard Fourier shell correlation (FSC) with the default C1 symmetry setting. The subsequent application of C3 symmetry in 3D refinement and a low-pass filter (25 Å) enhanced the map, achieving a global resolution of 3.12 Å at a FSC of 0.143 using 85,264 particles (Supplementary Fig. S4).

**Model building and refinement**

The model of UL78 trimer was built based on the cryo-EM structure of the US27−$G_i$−scFv16 (PDB code: 7RKX). The model was docked into the cryo-EM density map using UCSF Chimera v1.17.3, followed by iterative manual adjustment and rebuilding in COOT v0.9.8 and Phenix v1.20.1. Real-space refinement was performed using Phenix v1.20.1. The final refinement statistics were validated using the module comprehensive validation (cryo-EM) in Phenix v1.20.1. Structural figures were prepared with

UCSF ChimeraX v1.17, UCSF Chimera v1.17 and PyMOL v.2.1 (https://pymol.org/2/). The final refinement statistics are provided in Supplementary Table S2.

**Bimolecular fluorescence complementation–BRET assay**

To investigate GPCR oligomerization, a bimolecular fluorescence complementation-BRET (BiFC−BRET) method was adopted as previously reported[1-4]. In this approach, BiFC was utilized with a split EGFP, which fluoresces only when its amino- and carboxy-terminal fragments are brought closely together. By integrating BiFC with the BRET system, specific detection of the BRET signal is ensured upon the assembly of three molecules. For the experiment, UL78 expression vectors were engineered to carry enhanced GFP fragments (residues 1-144) and GFP fragments (residues 145-238) at their C termini, as well as fusion with Renilla luciferase (Rluc) at the C terminus. HEK293T cells were cultured in a 96-well plate and cotransfected with equal amounts of plasmids for UL78-EGFP(1-144), UL78-EGFP(145-238) and UL78-Rluc. Twenty-four hours post-transfection, cells were harvested and incubated with coelenterazine to facilitate the BRET reaction. The fluorescent and bioluminescent signals were subsequently measured using the EnVision system (PerkinElmer). BRET efficiency was quantified based on the ratio of emission at 535 nm to that at 480 nm. For validation, an EGFP-Rluc fusion protein was served as positive control to present optimal BRET efficiency. For negative control, cells were co-transfected with separate EGFP and Rluc expression plasmids which showed minimal BRET interaction.

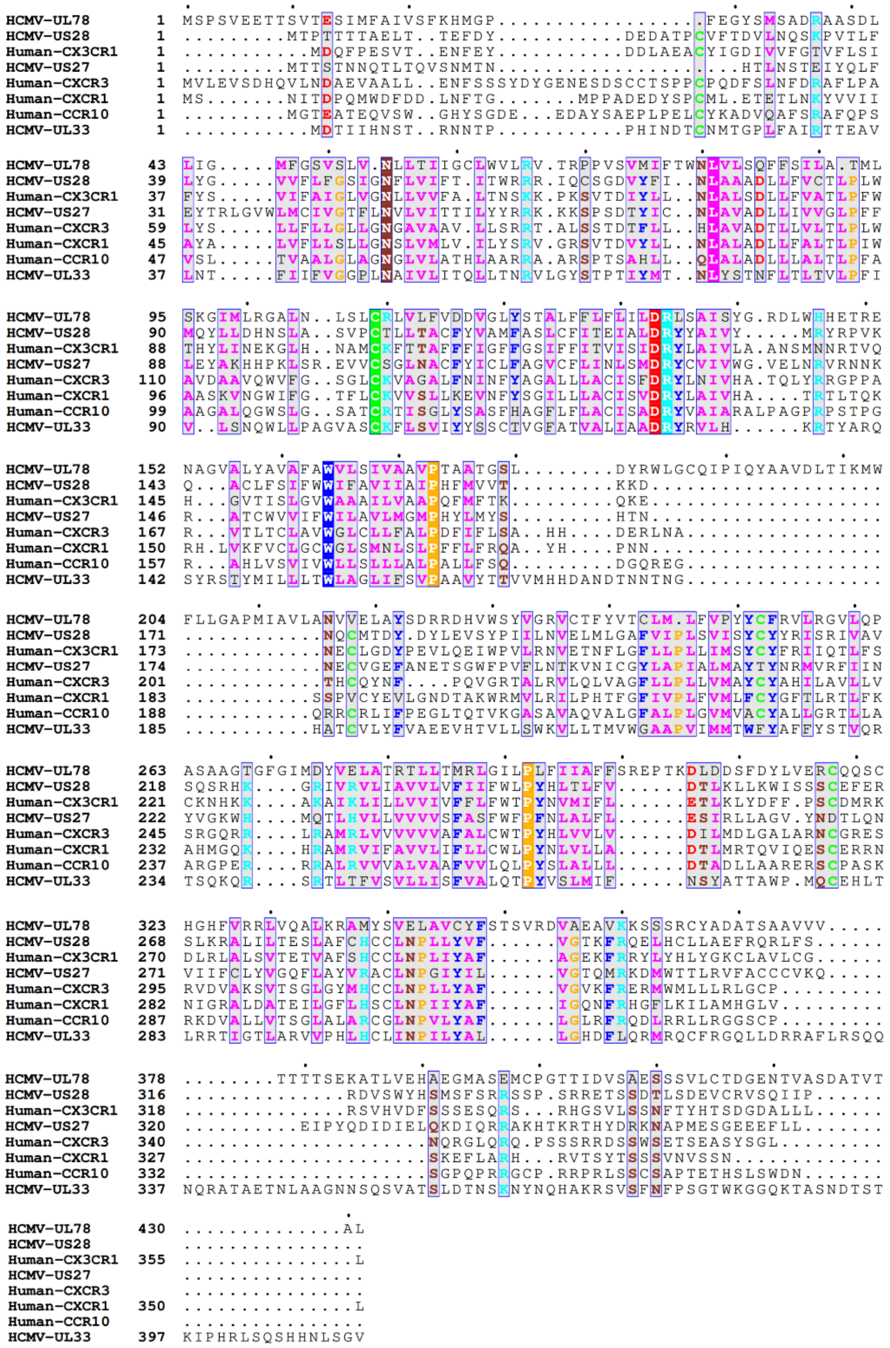


**Supplementary Fig. S1 Multiple sequence alignment of HCMV-encoded GPCRs (UL78, UL33, US28 and US27) and their homologous human receptors (CX3CR1, CXCR3, CXCR1 and CCR10).** The alignment of these sequences was conducted using the Multiple Sequence Alignment (MSA) and Espript 3.0 server. Residues are color-coded based on their physicochemical properties. All protein sequences were obtained from UniProt database.

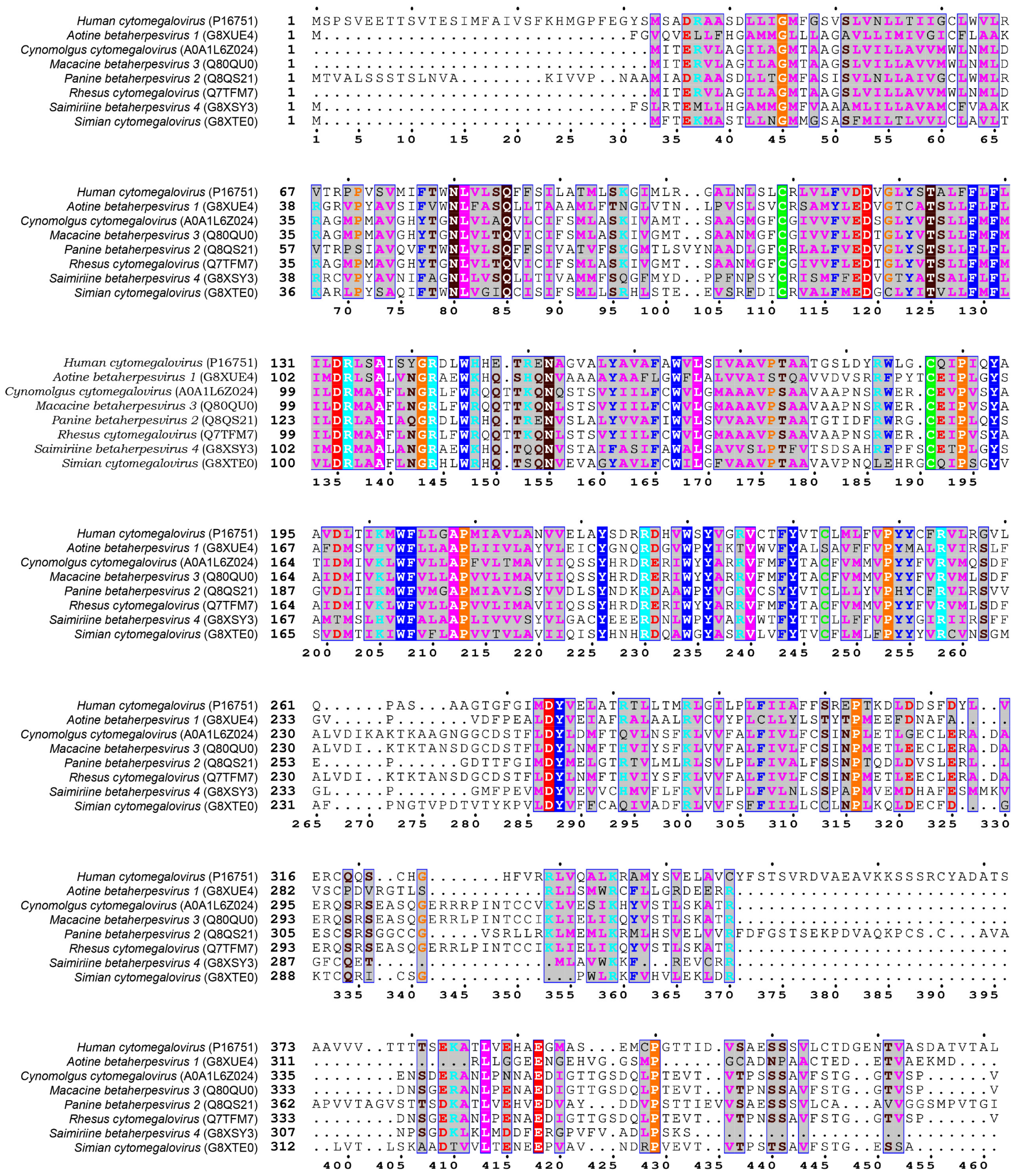

**Supplementary Fig. S2 Sequence conservation analysis of UL78 proteins across eight herpesvirus family members.** The UniProt accession codes (www.uniprot.org) are labeled in parentheses. Residues are colored by their physicochemical properties.

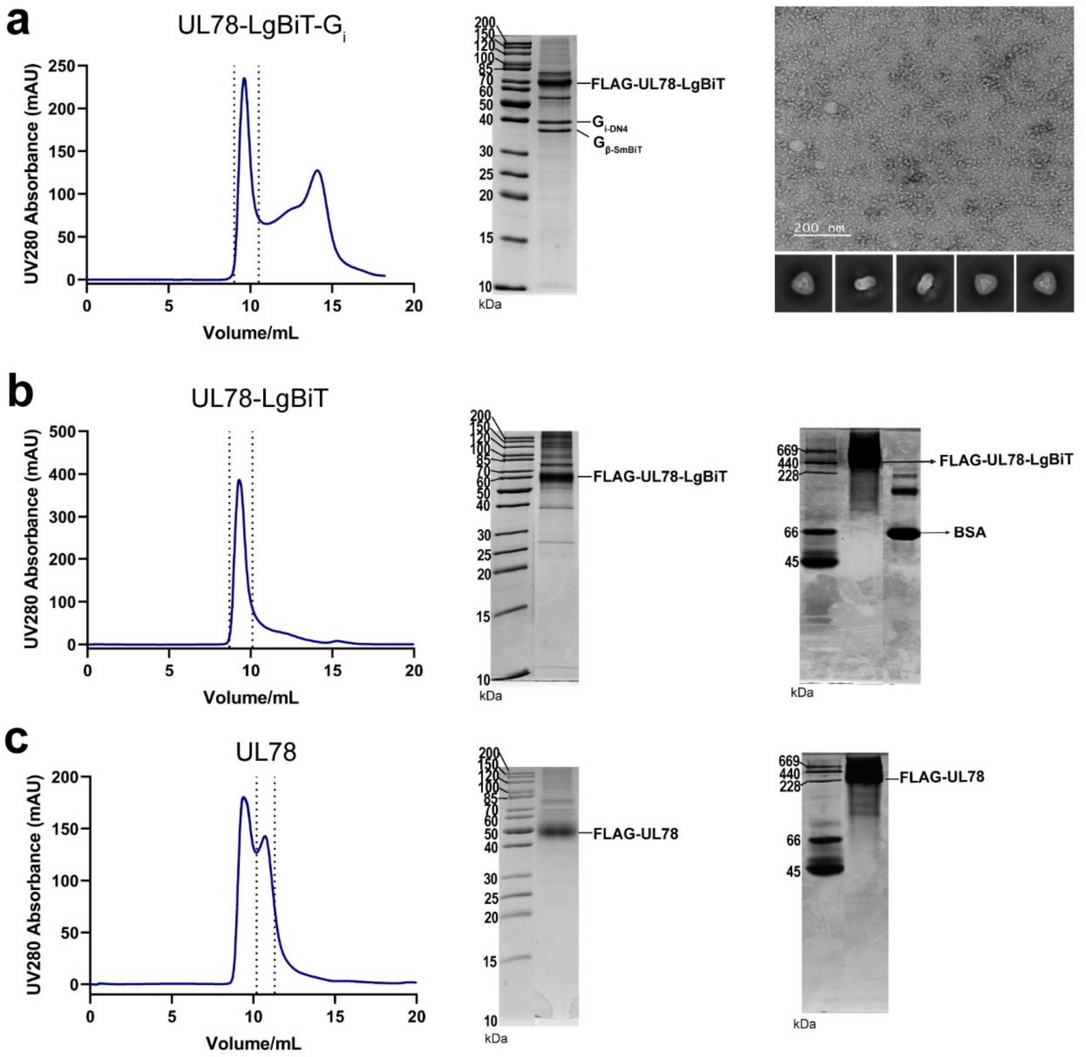


**Supplementary Fig. S3 Purification and cryo-EM data imaging of the UL78 trimer. a** Purification of the UL78−$G_i$ complex. Left, analytical size-exclusion chromatography; middle, corresponding SDS-PAGE/Coomassie blue; right, cryo-EM micrograph and representative two-dimensional class averages of the selected particles. **b, c** Purification of the UL78−LgBiT and UL78 indicates the formation of UL78 homotrimer in absence of $G_i$ protein. Left, analytical size-exclusion chromatography; middle, corresponding SDS-PAGE/Coomassie blue; right, Non-denaturing Protein Gel Electrophoresis/Coomassie blue of the UL78 trimer. The molecular weight of bovine serum albumin (BSA) is 66.5 kDa.

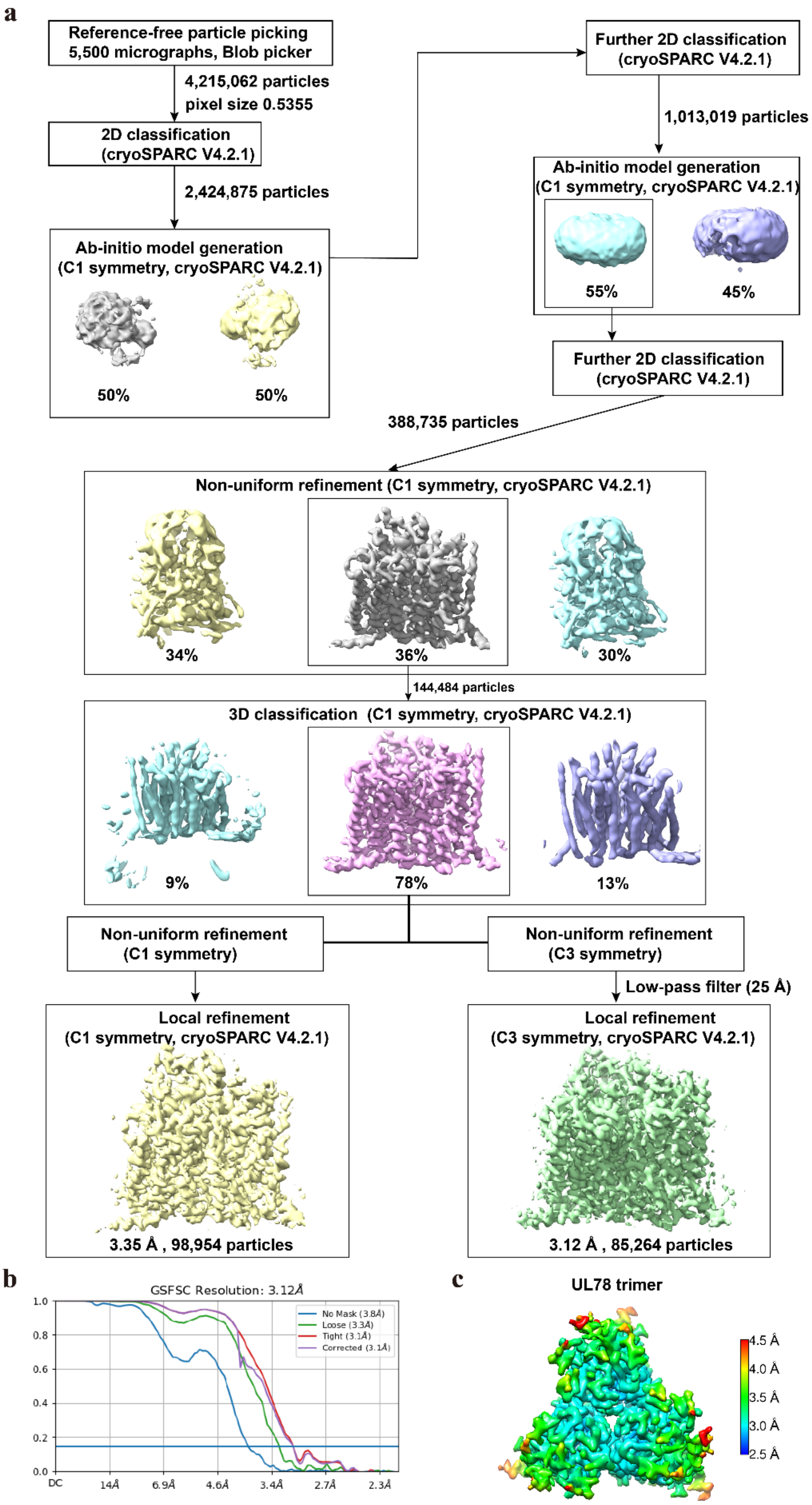


**Supplementary Fig. S4 Cryo-EM data processing of the UL78 trimer. a** Data processing of the cryo-EM dataset. Application of C3 symmetry in the 3D model reconstruction and a low-pass filter (25 Å) enhanced the map resolution from 3.35 Å to 3.12 Å. **b** The Fourier shell correlation curve of the density map. **c** Local resolution of the density map.

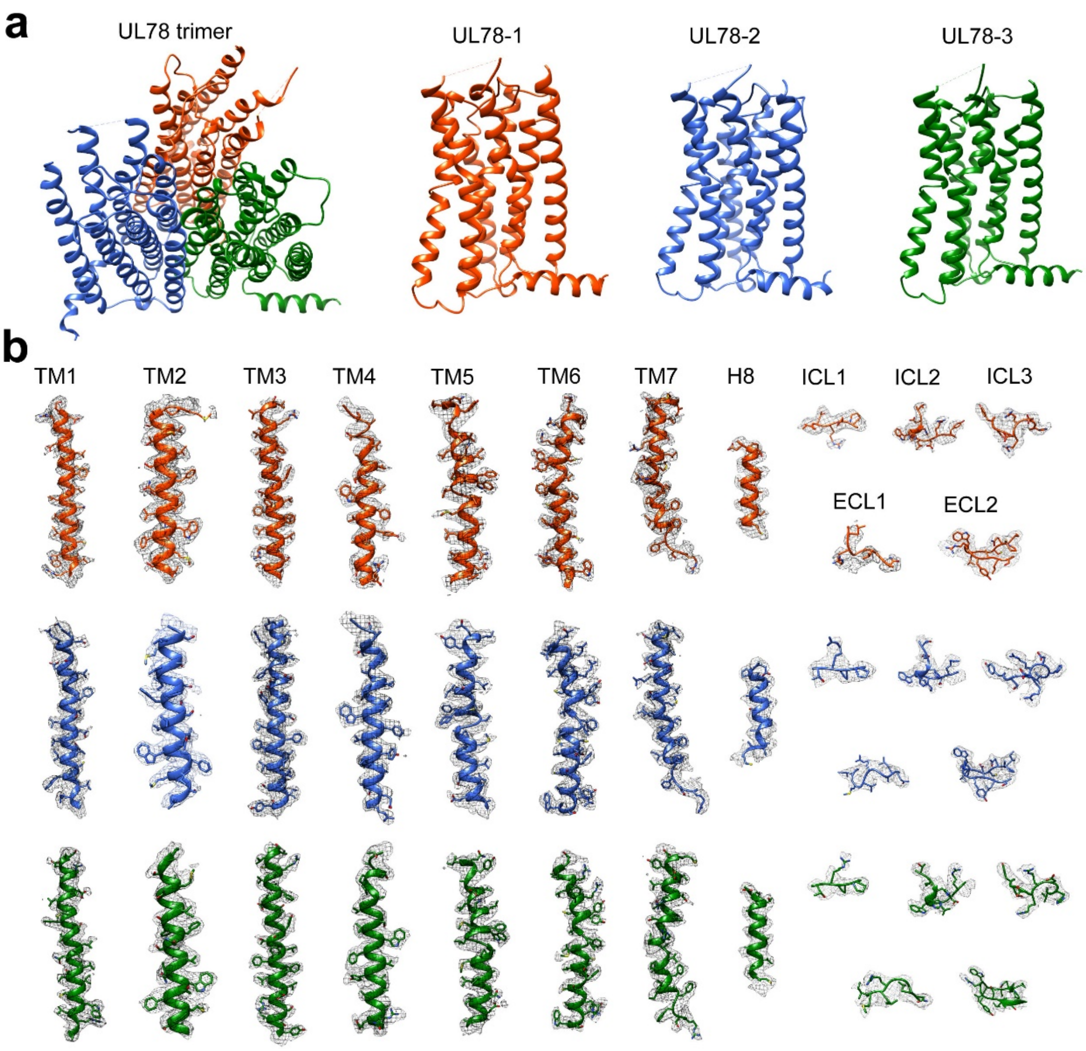


**Supplementary Fig. S5 Structure modeling of the UL78 trimer. a** Overall structure of the UL78 trimer. **b** The cryo-EM density maps and models are shown for all seven transmembrane helices (TMs), extracellular loops (ECLs) 1 and 2, and intracellular loops (ICLs) 1-3.

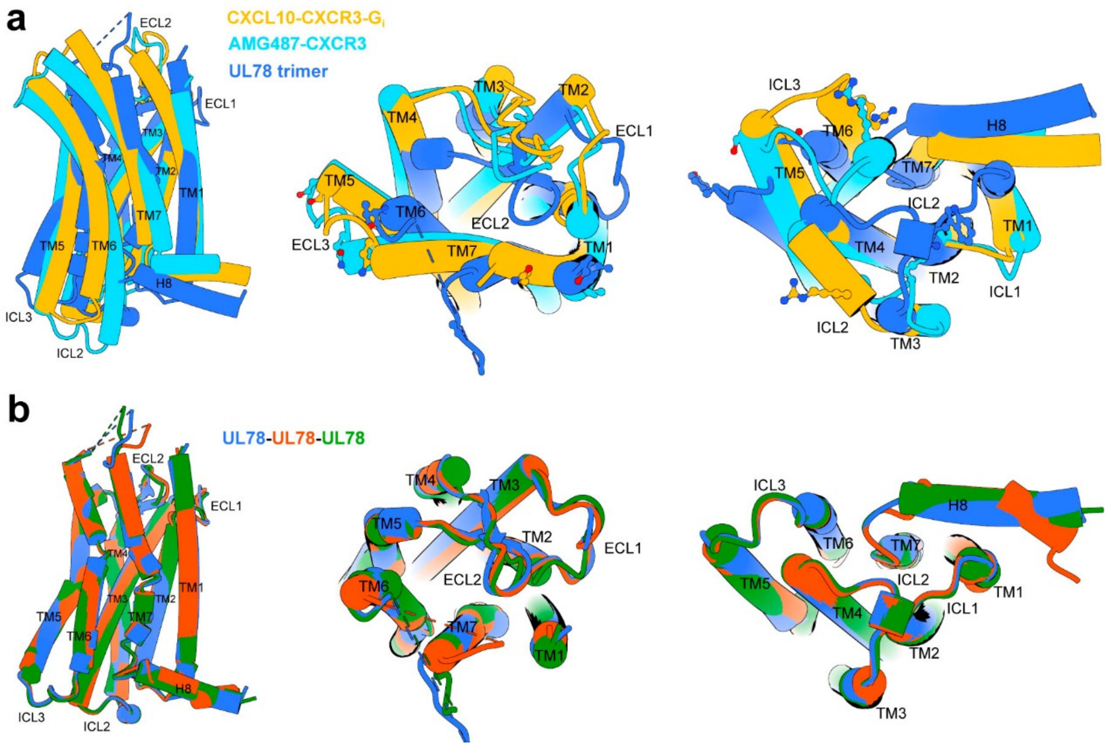


**Supplementary Fig. S6 Conformational comparison of the UL78 with active and inactive CXCR3.** **a** Superimposition of the UL78 and inactive state (PDB code: 8K2W) or active (PDB code: 8K2X) CXCR3 shows a unique closed conformation of UL78. **b** Superimposition of UL78 from the same trimer shows an undifferentiated conformation of each protomer.

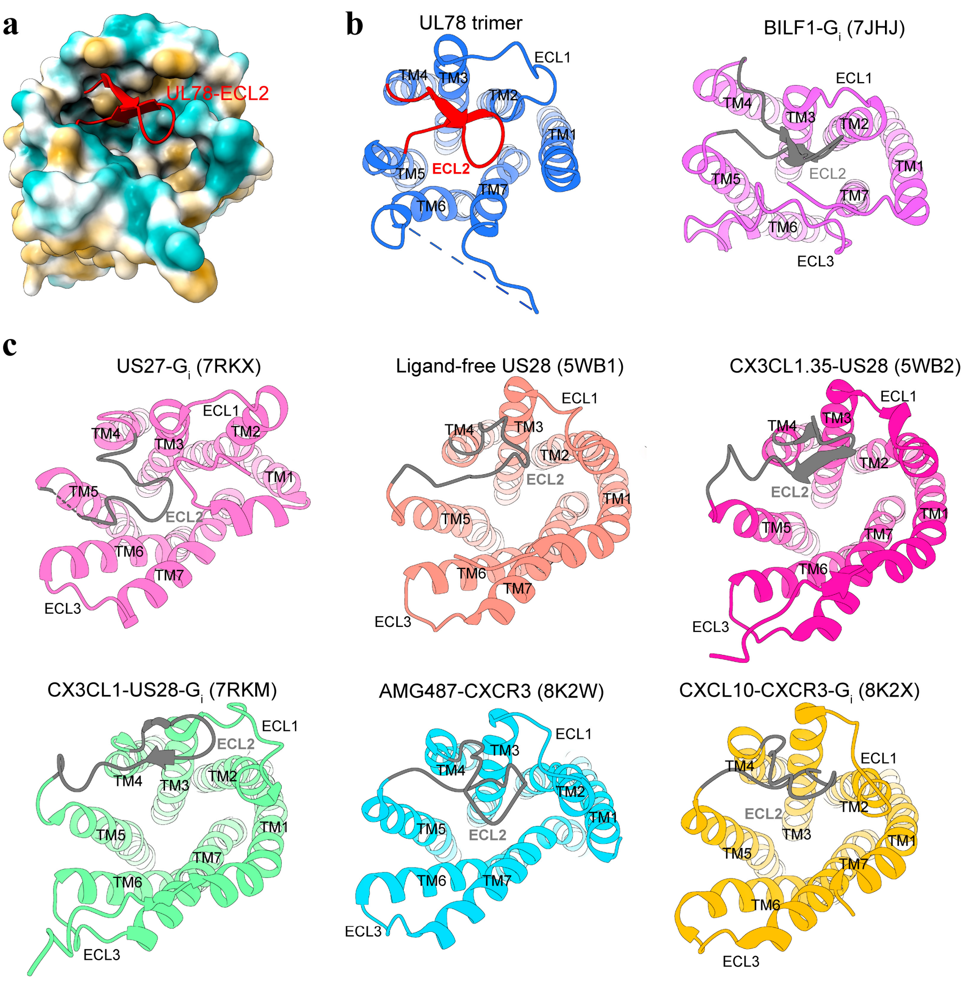


**Supplementary Fig. S7 Extracellular capping feature of the UL78. a** Top view of the UL78 structure. The transmembrane domain is shown as surface presentation and overlaid by the cartoon model of ECL2 (red). **b, c** Structural comparison of ECL2 among UL78 and other chemokine receptors. Cartoon representation of BILF1−$G_i$ (PDB code: 7JHJ), US27−$G_i$ (PDB code: 7RKX), ligand-free US28 (PDB code: 5WB1), CX3CL1.35-bound US28 (PDB code: 5WB2), CX3CL1−US28−$G_i$ (PDB code: 7RKM), AMG487-bound human CXCR3 (PDB code: 8K2W) and CXCL10−CXCR3−$G_i$ (PDB code: 8K2X) from the top view. The ECL2 regions are colored in red and gray for UL78 and other receptors, respectively.

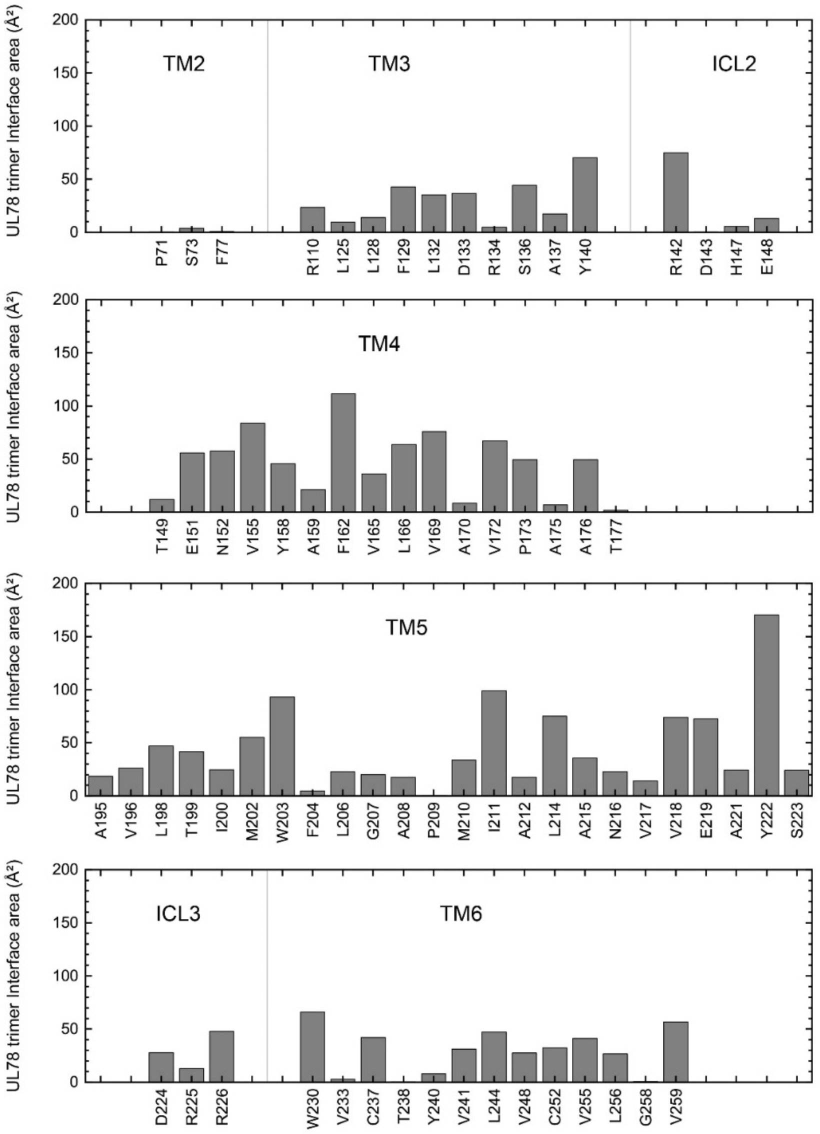


**Supplementary Fig. S8 Per-residue interface area decomposition for the UL78 trimer.** The interface area for each residue was measured with FreeSASA 2.0 by comparing the solvent-accessible surface area of the structure models of the UL78 homotrimer and its extracted UL78 protomer.

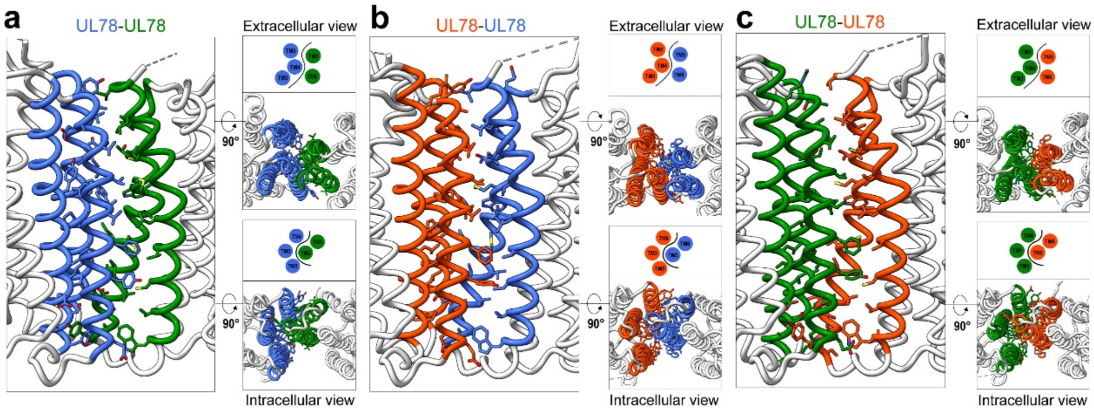


**Supplementary Fig. S9 Interfaces of the UL78 trimer.** Overall structure of the trimeric UL78 shows the interface of protomers 1 and 2 (**a**), 2 and 3 (**b**) as well as 1 and 3 (**c**). Extracellular and intracellular views show two interfaces composed of TM4, TM5 and TM6 from each protomer, with interface residues shown as sticks. UL78 protomers 1, 2 and 3 are colored in royal blue, dark green and orange red, respectively.

**Supplementary Table S1.** List of the reported virus-encoded GPCR structures.

| Receptor | PDB code | Method | Structural ligand | Intracellular binder | State | PDB title | Reference (PMID) |
|---|---|---|---|---|---|---|---|
| **US28** | 4XT1 | X-ray | CX3CL1 | Nanobody 7 | Active | Structure of a nanobody-bound US28 bound to human chemokine CX3CL1 | 25745166 |
| | 4XT3 | X-ray | CX3CL1 | None | Active | Structure of a viral GPCR bound to human chemokine CX3CL1 | 25745166 |
| | 5WB1 | X-ray | Apo (no ligand) | Nanobody 7 | Active | Ligand-free US28 with stabilizing intracellular nanobody | 29882741 |
| | 5WB2 | X-ray | CX3CL1.35 | Nanobody B1 | Active | US28 bound to engineered chemokine CX3CL1.35 and nanobodies | 29882741 |
| | 7RKF | Cryo-EM | CX3CL1 | $G_{11}$ | Active | Structure of CX3CL1-US28-G11iN18-scFv16 in TL-state | 35061538 |
| | 7RKM | Cryo-EM | CX3CL1 | $G_i$ | Active | Structure of CX3CL1-US28-Gi-scFv16 in C-state | 35061538 |
| | 7RKN | Cryo-EM | CX3CL1 | $G_i$ | Active | Structure of CX3CL1-US28-Gi-scFv16 in OC-state | 35061538 |
| **US27** | 7RKY | Cryo-EM | Apo (no ligand) | $G_i$ | Active | Binding mode of US27-Gi-scFv16 in OCL-state | 35061538 |
| | 7RKX | Cryo-EM | Apo (no ligand) | $G_i$ | Active | Structure of US27-Gi-scFv16 in CL-state | 35061538 |
| **BILF1** | 7JHJ | Cryo-EM | Apo (no ligand) | $G_i$ | Active | Structure of the Epstein-Barr virus GPCR BILF1 in complex with human Gi | 34216564 |

**Supplementary Table S2.** Cryo-EM data collection, refinement and validation statistics of the UL78 trimer.

| **Data collection and processing** | |
|---|---|
| Magnification | 46,685 |
| Voltage (kV) | 300 |
| Electron exposure ($e^{-}/Å^{2}$) | 80 |
| Defocus range (μm) | -1.2 to -2.2 |
| Pixel size (Å) | 1.071 |
| Symmetry imposed | CI |
| Final particle images (no.) | 85,264 |
| Map resolution (Å) | 3.12 |
| FSC threshold | 0.143 |
| **Refinement** | |
| Initial model used (PDB code) | 7RKX |
| Model resolution (Å) | 3.26 |
| FSC threshold | 0.5 |
| Model resolution range (Å) | 2.5–5.0 |
| Model composition | |
| Non-hydrogen atoms | 6,519 |
| Protein residues | 834 |
| B factors ($Å^{2}$) | |
| Protein | 86.23 |
| R.m.s. deviations | |
| Bond lengths (Å) | 0.002 |
| Bond angles (°) | 0.424 |
| Validation | |
| MolProbity score | 1.21 |
| Clash score | 4.33 |
| Poor rotamers (%) | 0.44 |
| Ramachandran plot | |
| Favored (%) | 98.78 |
| Allowed (%) | 1.22 |
| Disallowed (%) | 0 |

**Reference**


1 Cabantous, S. & Waldo, G. S. In vivo and in vitro protein solubility assays using split GFP. *Nat Methods* **3**, 845-854 (2006). https://doi.org/10.1038/nmeth932

2 Ip, D. T., Wong, K. B. & Wan, D. C. Characterization of novel orange fluorescent protein cloned from cnidarian tube anemone Cerianthus sp. *Mar Biotechnol (NY)* **9**, 469-478 (2007). https://doi.org/10.1007/s10126-007-9005-5

3 Hamatake, M. *et al.* Ligand-independent higher-order multimerization of CXCR4, a G-protein-coupled chemokine receptor involved in targeted metastasis. *Cancer Sci* **100**, 95-102 (2009). https://doi.org/10.1111/j.1349-7006.2008.00997.x

4 Rose, R. H., Briddon, S. J. & Holliday, N. D. Bimolecular fluorescence complementation: lighting up seven transmembrane domain receptor signalling networks. *Br J Pharmacol* **159**, 738-750 (2010). https://doi.org/10.1111/j.1476-5381.2009.00480.x
